# Predicting Survival in Glioblastoma Patients Using Diffusion MR Imaging Metrics—A Systematic Review

**DOI:** 10.3390/cancers12102858

**Published:** 2020-10-04

**Authors:** Valentina Brancato, Silvia Nuzzo, Liberatore Tramontano, Gerolama Condorelli, Marco Salvatore, Carlo Cavaliere

**Affiliations:** 1 IRCCS SDN (Istituto di Ricovero e Cura a Carattere Scientifico, SYNLAB istituto di Diagnostica Nucleare), 80131 Naples, Italy; valentina.brancato@synlab.it (V.B.); liberatore.tramontano@synlab.it (L.T.); direzionescientifica@sdn-napoli.it (M.S.); carlo.cavaliere@synlab.it (C.C.); 2 Department of Molecular Medicine and Medical Biotechnology, “Federico II” University of Naples, Via Tommaso de Amicis 95, 80131 Naples, Italy; gecondor@unina.it; 3 IRCCS Neuromed—Istituto Neurologico Mediterraneo Pozzilli, Via Atinense 18, 86077 Pozzilli, Italy

**Keywords:** glioblastoma, diffusion MRI, overall survival, progression free survival, DWI, DTI

## Abstract

**Simple Summary:**

An accurate survival analysis is crucial for disease management in glioblastoma (GBM) patients. Due to the ability of the diffusion MRI techniques of providing a quantitative assessment of GBM tumours, an ever-growing number of studies aimed at investigating the role of diffusion MRI metrics in survival prediction of GBM patients. Since the role of diffusion MRI in prediction and evaluation of survival outcomes has not been fully addressed and results are often controversial or unsatisfactory, we performed this systematic review in order to collect, summarize and evaluate all studies evaluating the role of diffusion MRI metrics in predicting survival in GBM patients. We found that quantitative diffusion MRI metrics provide useful information for predicting survival outcomes in GBM patients, mainly in combination with other clinical and multimodality imaging parameters.

**Abstract:**

Despite advances in surgical and medical treatment of glioblastoma (GBM), the medium survival is about 15 months and varies significantly, with occasional longer survivors and individuals whose tumours show a significant response to therapy with respect to others. Diffusion MRI can provide a quantitative assessment of the intratumoral heterogeneity of GBM infiltration, which is of clinical significance for targeted surgery and therapy, and aimed at improving GBM patient survival. So, the aim of this systematic review is to assess the role of diffusion MRI metrics in predicting survival of patients with GBM. According to the Preferred Reporting Items for Systematic Reviews and Meta-Analyses (PRISMA) statement, a systematic literature search was performed to identify original articles since 2010 that evaluated the association of diffusion MRI metrics with overall survival (OS) and progression-free survival (PFS). The quality of the included studies was evaluated using the QUIPS tool. A total of 52 articles were selected. The most examined metrics were associated with the standard Diffusion Weighted Imaging (DWI) (34 studies) and Diffusion Tensor Imaging (DTI) models (17 studies). Our findings showed that quantitative diffusion MRI metrics provide useful information for predicting survival outcomes in GBM patients, mainly in combination with other clinical and multimodality imaging parameters.

## 1. Introduction

Glioblastoma (GBM) is classified by World Health Organization as a grade IV astrocytoma and is the most common and fatal primary brain tumour of the central nervous system (CNS) in adults [1]. GBMs can be classified based on their clinical history as primary (pGBM) or secondary (sGMB). pGBMs are the most common (about 90%) and are characterised by the absence of a clinical or pathologic evidence of a less malignant precursor, while sGBMs result from lower grade gliomas that evolved into GBM. pGBM patients tend to be older and have a poorer prognosis than patients with sGBMs [2].

The clinical presentation and symptoms of a patient with a newly diagnosed GBM are highly variable and dependent on the size, location and degree of infiltration of the tumour [3].

The current clinical treatment for newly diagnosed GBM requires a multidisciplinary approach and consists of maximal tumour resection, followed by concurrent chemoradiotherapy with temozolomide (CCRT) and adjuvant temozolomide (TMZ) [4]. However, despite maximal safe surgical resection and multimodality therapy, about 70% of these tumours invariably recurs, with standards of care at recurrence far less well defined than in the newly diagnosed setting [3,5,6]. Despite advances in surgical and medical neuro-oncology, the medium survival is only 15 months after first diagnosis and with standard surgery and chemoradiation treatment [3,7,8,9,10]. However, the overall survival of patients with GBM varies significantly and there are occasional longer survivors and individuals whose tumours show a significant response to therapy with respect to others [11,12]. For this reason, an accurate survival analysis is crucial to support surgical and therapeutic decisions and so for disease management in GBM patients. This has led to the search for various markers which could be predictive of patient outcome or treatment response [11]. Several clinical (e.g., patient age at diagnosis and Karnofsky Performance Status (KPS)) and therapeutic (extent of surgery, radiation therapy and chemotherapy) factors, as well as specific tumour characteristics (e.g., volume and location) and histopathological and genetic markers (Ki67, isocitrate dehydrogenase 1—IDH1—mutation status and O6-methylguanin-DNA-methyltransferase—MGMT—promoter methylation status) have been studied as potential prognostic markers of survival with variable degrees of sensitivity and specificity [12].

However, their assessment currently requires tissue removing through biopsy or surgical procedures. Thus, a non-invasive assessment of GBM prognosis would be particularly important for treatment planning and GBM patient management in the context of personalized medicine. In this scenario, imaging represents an attractive option compared to more invasive approaches based on tissue-derived biomarkers. There is an urgent need to discover imaging biomarkers that can aid in the selection of patients who will likely derive the most benefit from surgical and/or chemotherapy and/or radiotherapy treatment in terms of overall survival (OS) and progression-free survival (PFS), which are the most commonly evaluated clinical endpoints [13]. Conventional magnetic resonance imaging (MRI) employed for GBM investigation include T1-weighted imaging (T1WI), T2WI, fluid attenuation inversion recovery (FLAIR), T2*WI gradient echo and contrast-enhanced T1WI. These sequences can yield information on the gross anatomic structure of GBM, but they provide little functional information. In fact, the poor prognosis of GBM patients is largely attributed to tumour growth and infiltration that are sometimes difficult to detect by conventional MRI, making novel imaging biomarkers important for aiding in both tumour spatial localization and patient survival prediction [14,15,16].

Given the lack of standardization for their use in GBM clinical practice, advanced imaging techniques such as Positron Emission Tomography (PET), in which different radiotracers are injected to target metabolic and molecular profiles, Perfusion Weighted Imaging (PWI), which provides information on tissue perfusion and microvascular permeability, and diffusion MRI techniques were investigated for GBM management [17,18,19,20].

Diffusion MRI-derived parameters can provide a quantitative assessment of the intratumoral heterogeneity of GBM infiltration, adding new microstructural insights on apparent normal peritumoral white matter that are not detectable by conventional MRI. So, there is increasing interest in these techniques as a biomarker of prognosis in GBM patients. 

Diffusion Weighted Imaging (DWI) and Diffusion Tensor Imaging (DTI) are the most commonly used diffusion techniques for GBM applications. In particular, they hold great promise for the improvement of GBM diagnosis and identification of histological classification, prediction of treatment response and monitoring GBM recurrence [20,21,22]. DWI is sensitive to water proton motion at the cellular level and can provide information concerning the microscopic structural environment of neoplasm [21,23]. DTI, other than measuring the magnitude of water molecule movement, such as DWI, is also able to quantify the orientation of the diffusion and has been shown to be sensitive in detecting tumour infiltration [21,23,24]. 

However, more advanced diffusion imaging techniques, such as non-Gaussian diffusion techniques (e.g., Diffusion Kurtosis Imaging (DKI), Intravoxel Incoherent Motion (IVIM), Stretched Exponential (SE) and Restriction Spectrum Imaging (RSI) were also investigated for GBM applications [25,26,27,28].

Given the urgent need for finding the optimal and targeted treatment option for GBM patients [29,30], and given the proven power of diffusion MRI techniques for applications such as preoperative grading, postoperative assessment of glial tumours and differentiation of GBM from other tumours types (such as brain metastasis or PCNSL [31,32,33]), several studied investigated the association of diffusion MRI metrics with survival outcomes in patients with GBM. Some of these were performed on newly diagnosed GBM patients, while others on recurrent GBM patients. A wide range of diffusion metrics arising from different diffusion MRI models (DWI, DTI, non-Gaussian DWI models and RSI) were explored.

However, their role in prediction and evaluation of survival outcomes has not been fully addressed and results are often controversial or unsatisfactory.

In the context of precision medicine, the analysis of OS and PFS in patients receiving a certain surgical treatment and/or therapy for newly diagnosed or recurrent GBM may help clinicians to better understand the evolution of this disease in each single patient, thus improving patient care and clinical decision-making. Therefore, the aim of this systematic review is to collect, summarize and discuss all studies evaluating the role of diffusion MRI metrics in predicting survival in GBM patients and raise awareness for future research in this field.

## 2. Materials and Methods

### 2.1. Search Strategy and Selection Criteria

A systematic search for all the published studies examining the association of diffusion metrics arising from any diffusion model with OS and/or PFS was conducted. The most relevant scientific electronic databases (PubMed, Cochrane Library, MEDLINE, ScienceDirect and Google Scholar) were comprehensively explored and used to build the search. Only studies published since 2010 were selected. The search strategy included the key terms listed in Supplementary Materials Table S1. The literature search was restricted to English language publications and studies of human subject. 

Two reviewers, after having independently screened identified titles and abstracts, assessed the full text of the articles that evaluated at least one diffusion MRI metric in terms of OS or PFS, and were not review articles.

For articles meeting these criteria with full text available, the following further selection criteria had to be fulfilled: adult patients; patients with histopathologically confirmed GBM (newly diagnosed or recurrent); and at least one diffusion MRI metric examined in terms of OS or PFS. Studies were excluded if the patient population included also patients with any type of brain tumours other than GBM.

### 2.2. Planning and Conducting the Review

After the selection procedure, the selected articles were analysed by two reviewers, and data useful for conducting the systematic review were collected in a predesigned sheet. The extracted data will include the following: study characteristics (first author name, publication year and study design, in particular, prospective/retrospective and cross-sectional/longitudinal and number of patients); patient characteristics (age, diagnosis and treatment); MR examination timepoints, namely, the MR images from which the investigated diffusion metrics derive; clinical outcome (OS and/or PFS); diffusion MRI model/s evaluated; diffusion acquisition details; diffusion MRI metric/s evaluated; information on placement of regions of interest (ROIs), namely, the segmentation method and MRI sequence on which the ROIs were placed on; performing the statistical analysis; and the main findings.

The articles were classified and analysed according to the diffusion model involved and the diagnosis (newly diagnosed GBM or recurrent). If more than one diffusion model was investigated in the same study, each model was treated as belonging to a separated study.

This systematic review was conducted in accordance with the Preferred Reporting Items for Systematic Reviews and Meta-Analyses (PRISMA) statement (see Supplementary Materials Table S2 for the PRISMA checklist).

### 2.3. Quality Assessment

The quality of the individual studies was assessed using the QUality In Prognosis Studies (QUIPS) tool [34,35]. According to QUIPS, six domains are critical for assessing biases in prognostic studies: selection of study participants, study attrition, prognostic factor measurement, outcome measurement and study confounding and statistical analysis and reporting. For each of these six domains, the responses “yes”, “partial”, “no” or “unsure” for three up to seven items within each domain are combined to assess the risk of bias. An overall rating for each domain is assigned as “high”, “moderate” or “low” risk of bias. The QUIPS assessment for each study was independently completed by two reviewers and discrepancies were resolved by discussion.

## 3. Results

### 3.1. Study Selection

**Figure 1:**
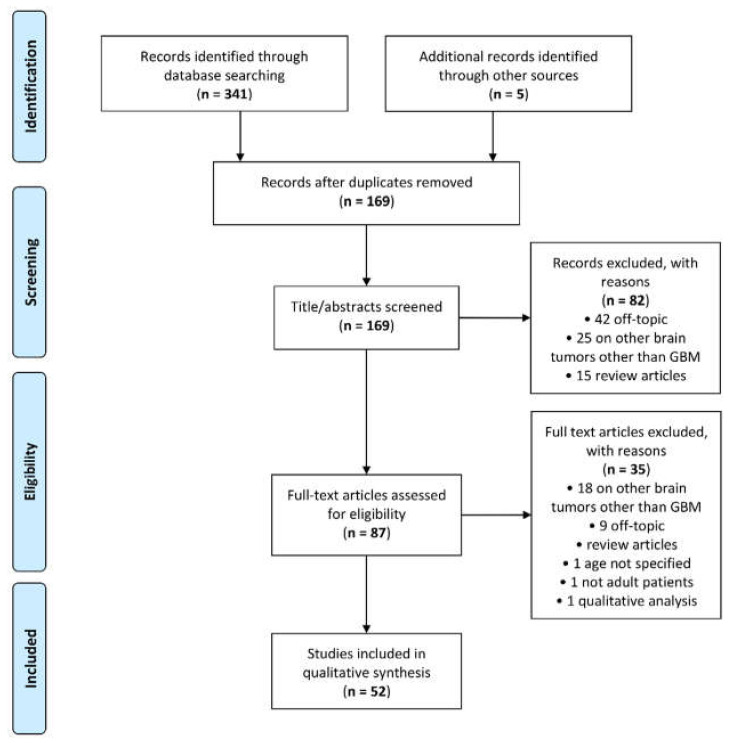
Preferred Reporting Items for Systematic Reviews and Meta-Analyses (PRISMA) flow diagram.

A total of 341 articles was retrieved by the scientific electronic databases search. Five additional articles were found through article references, bringing the total number of records suitable for further evaluation to 346. After removal of the duplicates, there were 169 articles left for investigation. By scanning the title and abstract of these records, 82 records were excluded because they clearly did not match the inclusion criteria (15 review articles, 42 off-topic and 25 including patients with other brain tumours other than GBM). A total of 87 articles were evaluated on their full text. Of these articles, 35 records were excluded based on the inclusion criteria (1 was excluded because it involved non-adult patients [36]; 1 was excluded because it did not specify patient age [37]; 1 was excluded because it performed prognostic analysis based on qualitative visual examinations [38]; 5 were excluded since they were review articles; 18 were excluded since they involved also other types of brain tumour other than GBM; and 9 were not in the field of interest). Finally, 52 records were included for qualitative synthesis. The PRISMA flow diagram of the included studies according to the inclusion and exclusion criteria is reported in Figure 1.

### 3.2. Characteristics of the Included Studies

The study characteristics of the 52 articles selected for this review are described in Table 1. 

All the selected studies were targeted to adults and the median number (±absolute deviation) of individuals was 53 ± 44.27. Most study designs were retrospective (43/52).

Most of the selected papers (39 studies) involved patients with newly diagnosed GBM (ND-GBM), while 13 studies were specific for recurrent GBM (R-GBM). However, it should be highlighted that in studies involving ND-GBM patients, it is frequently so that several patients present recurrence during clinical history (until death in case of OS analysis). Concerning patient treatment, in studies on ND-GBM patients, most patients received the standard treatment, including chemotherapy and radiotherapy after surgical resection. Among studies on R-GBM patients, only two study involved patients who underwent a second surgery [39,40], while the remaining 11 involved patients receiving chemotherapy and/or radiotherapy treatments. However, many studies did not refer to a uniform treatment for all patients though TMZ and bevacizumab (BV) were the most used chemotherapeutic drugs. See Table 1 for more details on treatments of patients.

Management timepoints at the MR examination vary across studies. Concerning ND-GBM patients, most studies used the preoperative study setting (26/39), while the remaining study investigated metrics arising from postoperative MRI examinations, often considering multiple timepoints. All studies on R-GBM patients investigated the parameters arising from pretreatment and/or posttreatment after recurrence.

**Figure 2:**
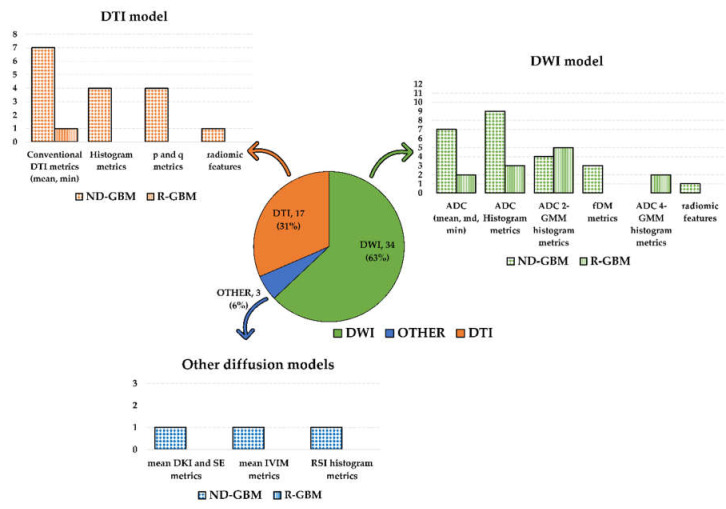
Graphic summary of the systematic review results according to the diffusion models and metrics investigated in the selected studies. The pie chart shows the number of included studies according to the diffusion MRI model (DWI in green; DTI in orange; other diffusion models in blue). Number and percentage of studies included in each of the three groups were reported. The bar plots show the diffusion metrics investigated in each group. Dotted bars count studies on newly diagnosed GBM patients, while the bars filled with vertical lines count studies on recurrent GBM patients. The study by Wen et al. [55] was counted three times in the DWI model histogram since they investigated both histogram and ADC 2-GMM and fDM metrics.

Most of the selected papers examined metrics associated with the standard DWI model (34 studies). The second most examined metrics were associated with the DTI model (17 studies). Among the studies investigating the DWI model, one investigated DWI and RSI [28], while one DWI together with DKI and SE [26]. The remaining study investigated the IVIM model [25]. Refer to Figure 2 for a graphic visualization of the obtained results according to the diffusion models and metrics investigated in the selected studies.

### 3.3. Association of Diffusion MR Imaging Metrics with OS and PFS

#### 3.3.1. DWI Metrics

A total of 34 studies investigated the association of DWI metrics with survival endpoints. Among them, 22 involved ND-GBM patients, while the remaining 12 concern R-GBM patients. Concerning studies involving ND-GBM patients, several of them investigated the power of the ADC mean, median and minimum (min) values in tumoral regions, sometimes considering also its normalized value for the normal-appearing white matter (NAWM) or normal contralateral brain [12,26,44,50,53,56,63]. Romano et al. [44] found that the min ADC values in enhancing the tumour component on T1-weighted images were associated with PFS and OS and so could be used as a preoperative parameter to predict patient survival. In particular, patients with higher ADC min values survived longer than patients with lower ADC min values. Similar results were found by Nakamura et al. [50] and Shankar et al. [63]. However, the latter considered only OS as a survival outcome and normalized the ADC min value as the diffusion metric, which showed a positive correlation with OS. Moreover, according to the survival analysis performed by Elson et al. [56] in the post-operative, pre-radiotherapy setting, the normalized ADC min associated with the hyperintense T2/FLAIR regions was a strong predictor of PFS and OS in patients with ND-GBM. Conversely, Coban et al. [12] found no significant difference in the min ADC between patients having a short and long OS. Omuro et al. [53] found that preoperative lower normalized ADC in the maximal portion of the T1 contrast-enhancing tumour was associated with prolonged OS, but not PFS. In Chakoyan et al. [26], patients with large, positive changes in their post-treatment median ADC have a higher OS compared to stable or decreasing ADC changes. Other studies on ND-GBM patients investigated the association with OS and PFS of the ADC histogram metrics (normalized or not) obtained performing standard histogram analysis [28,41,48,55,57,62,64,75,76] or using a double Gaussian mixture modelling for a histogram of the ADC intensities within the considered region [42,55,61,69]. Among the studies investigating the standard ADC histogram parameters, six studies investigated the association of the standard ADC histogram metrics with survival outcomes in a postoperative setting [28,41,55,57,64,76]. Li et al. [41], in a postoperative setting, found that the median and 10th percentile of the normalized ADC values observed pre-and post-treatment were not associated with OS and PFS in a time-independent analysis, but they were significantly correlated with OS according to a time-dependent survival analysis. Wen et al. [55] found an association between the ADC 10th and 50th percentile with both OS and PFS within the T2-enhanced lesions at 2 months post treatment, while in a study by Lee et al. [57], any association with PFS was found between the normalized ADC histogram metrics within the T1 contrast-enhancing lesions. Unfavourable results were also found by Van der Hoorn et al. [64], who evaluated the association of the histogram parameters of the post-treatment changes in normalized ADC within the periresectional area with OS and PFS. In this study, the increase in ADC value post-treatment in comparison to pre-treatment did not predict an increase in PFS or OS neither in the univariate nor multivariate survival analysis. Krishnan et al. [28] also found no association of ADC histogram metrics within the T1 contrast-enhanced and T2 FLAIR hyperintensity volume with OS and PFS. The remaining four studies investigating the power of the standard ADC histogram parameters for predicting survival in ND-GBM patients were performed in a preoperative setting [48,62,75,76]. In a study by Sunwoo et al. [48], PFS was positively associated with the mean ADC value in enhancing the tumour volumes segmented on the postcontrast T1-weighted presurgical scans, but not with the ADC 5th percentile in these regions. The univariate survival analysis performed by Burth et al. [62] showed a positive association of the ADC 10th percentile in T1-enhancing tumour volumes and hyperintense signal changes on T2 FLAIR with OS, but not with PFS. However, in multivariable analysis, the diffusion-derived MRI parameters did not predict survival. In a study by Kim et al. [76], lower ADC histogram parameters were significant predictors of poor OS. In a longitudinal study by Rulseh et al. [75], the ADC histogram analysis was performed using a whole-brain approach. Results showed that serial standardized median ADC and p85 values correlated with PFS and OS, respectively. Four studies investigated the survival prediction power of the histogram metrics obtained applying double-Gaussian mixture modelling (2-GMM) to ADC histograms in regions of interest [42,55,61,69]. A study by Pope et al. [42] performed on preoperative ADC images showed that the ADC mean values for the lower peak (ADCL) of the 2-GMM distribution can stratify PFS in ND-GBM patients with newly diagnosed GBM treated with BV. Moreover, although no statistically significant findings were found, the BV-treated patients with low ADCL also tended to have better OS. In the preoperative study by Kondo et al. [69], promising results also were reported on the association between broader and lower values in the ADCL and PFS and OS. The remaining two studies were performed in a postoperative setting and showed conflicting results [55,61]. Chang et al. ND-GBM patients with low ADCL after treatment have shorter PFS and OS than those with higher ADCL (within contrast-enhancing tumour regions on T1 subtraction images). The ADC mean values for the higher peak (ADCH) of the 2-GMM were not useful for stratifying survival. Conversely, according to Wen et al. [55], no ADC parameters from the 2-GMM fitting in T1 contrast-enhancing lesions were found to be associated with either OS or PFS.

Three studies investigated the power of functional diffusion maps (fDMs)-associated parameters for stratifying survival in ND-GBM patients [43,49,55]. Two of these were by Ellingson et al. [43,49] and showed that the fDMs and probabilistic fDMs metrics obtained considering pre- and post-treatment ADC maps were promising PFS and OS predictors. In a study by Wen et al. [55], the fDM metrics were associated with OS and PFS within the T1 contrast-enhancing lesions, but not in the T2-enhanced lesions. Only Park et al. [83] used a radiomic approach for building a multiparametric MR model able to predict OS in ND-GBM patients and incorporating ADC histogram skewness in its equation.

Concerning studies involving R-GBM patients, all but two studies [39,40] evaluated the predictive value of the ADC metrics in stratifying PFS and OS for R-GBM patients following a certain radio- and/or chemo-therapeutic treatment after the occurrence of first or second recurrence. The most investigated chemotherapeutic regimen was BV. Only two studies [39,40] investigated the ADC metrics survival prediction power for R-GBM patients undergoing a second surgery. Most of them used ADC metrics arising from the histogram analysis based on fitting a double-component Gaussian mixture model to the obtained ADC values [40,45,52,70,77]. In particular, Pope et al. [45] found that low ADCL within tumour regions placed on pre-treatment contrast-enhancing T1-weighted images at baseline was associated with worse OS and PFS in BV-treated R-GBM patients. Similar results were obtained by Ellingson et al. [52]. Differently, Buemi et al. [77] found ADCL in T1 contrast-enhancing tumour regions useful only for stratifying PFS, but not OS in R-GBM patients prior to BV treatment. In the same study, ADCL in T2/FLAIR abnormalities was unable to stratify both PFS and OS. In a subsequent study by Ellingson et al. [70], ADCL was predictive of OS in patients with recurrent GBM treated with anti-VEGF monotherapy at first or second recurrence. Patel et al. [40] also investigated the utility of ADCL, concluding that patients with low ADCL have a survival benefit when surgically excised, whereas large tumours with high ADCL may be best treated with BV. Three studies investigated the survival predictive power of the ADC monomodal histogram parameters [39,58,78], of which two involved BV-treated patients [58,78]. In the first it was found that, unlike the normalized ADC 5th percentile that was also evaluated, the volume of low-ADC lesions in enhancing pre- and post-BV scans predicted shorter OS [58], while in the second [78], using a machine learning approach, the ADC histogram metrics in combination with perfusion metrics arising from pre- and post-treatment images were found to be useful for BV response assessment in terms of PFS and OS. Among the histogram features inspected by Zolal et al. [39] in R-GBM patients undergoing their second surgery, the ADC histogram skewness was an independent prognostic factor for OS and PFS after the second surgery.

Two studies [54,65], rather than using a mono or bi-component curve-fitting histogram analysis as done in the previously described studies, used a four-component one. Rahman et al. [54] evaluated ADC within the volume of contrast-enhancing and non-enhancing T2/FLAIR lesions in BV R-GBM patients, revealing a significant association between the histogram parameters and OS and PFS after performing uni- and multivariable Cox-regression analyses. Chang et al. [65], in a similar study setting, used machine learning approach based on a multimodal approach, including ADC texture and four-component histogram metrics, and developed a valid predictive model for OS. The remaining two studies on R-GBM patients investigated the power of normalized ADC mean and min values in enhancing and non-enhancing tumoral regions [71,84]. Galla et al. [71] found that in R-GBM patients treated with superselective intra-arterial cerebral infusion of BV, the change in normalized ADC mean and min after treatment is predictive of OS. Song et al. [84] found that normalized ADC can be used as an imaging biomarker to determine PFS in R-GBM patients treated with immune checkpoint inhibitors.

#### 3.3.2. DTI Metrics

A total of 17 studies investigated the association of DTI metrics with survival endpoints, of which only 1 involved R-GBM patients, while the remaining involved ND-GBM patients. Amongst the latter, seven studies investigated the power of the mean and min values of diffusion tensor metrics in the tumoral regions, sometimes considering also their normalized value for normal-appearing brain tissue, in a preoperative setting [11,47,59,67,73,79,81]. The most investigated features were mean diffusivity (MD) and fractional anisotropy (FA). Jamjoom et al. [59] found that lower min MD within enhancing tumour regions predicted shorted OS. Similar behaviour was observed in a study by Heiland et al. [73] for mean MD. Conversely, the mean MD and values were found not predictive for OS in studies by Huber et al. and Mohan et al. [67,81] and, normalized for normal contralateral brain tissue, predictive for OS only in the univariate analysis performed by Zikou et al. [47], but not in the multivariate one. Controversial results were also observed concerning FA. In a study by Heiland et al. [73], a lower FA in the contrast-enhancing region was significantly associated with better OS. Moreover, Flores-Alvares et al. [79] found significant association between the peritumoral oedema measurement of FA with OS. Mohan et al. [81] found that FA in corpus callosum infiltrations was able to be a prognostic marker for prediction of OS in ND-GBM patients. On the other hand, in studies by Saksena et al. and Huber et al. [11,67], respectively considering tumour on FLAIR signal abnormality and contrast enhancing zones, FA showed promising results in terms of PFS and OS prediction only in univariate, but not multivariate survival analysis. Finally, although there is a detected trend towards better OS for patients with lower FA values, no significant results were found by Zikou et al. [47].

Four studies performed histogram analysis of DTI metrics [59,60,66,74]. In a preoperative setting, higher MD gradient histogram values of the tumour boundary predicted shorter OS in a study by Jamjoom et al. [59]. Promising results in terms of prediction of both OS and PFS were also showed by Choi et al. [66]. Specifically, lower MD histogram parameters were significant predictors of poor OS and PFS, according to univariate analysis. Multivariable models with MD parameters had significantly higher performances that those without MD parameters for OS and PFS prediction. However, conflicting results were obtained in a later study performed by the same group [74] since any feature from the ADC histogram parameter were useful to build a predictive model based on MRI radiomic features and clinical and genetic parameters, which lead to better performance in terms of OS and PFS when compared with models containing clinical and genetic profiles alone. In a postoperative setting, Wen et al. [60] compared the survival predictive power of the DTI histogram metrics in two differently treated cohorts, showing MD and radial eigenvalues association with OS and PFS at different timepoints. Moreover, in the same study, a volumetric analysis on MD low values was performed and volumetric diffusion parameters were also associated with OS and PFS at different timepoints. A volumetric analysis on MD low values was also performed in a study by Boonzaier et al. [72], who found that regions of low MD (implying high cellularity) and high relative cerebral blood volume (rCBV) (implying vascularity) were correlated with PFS and OS in non-enhancing GBM regions.

The remaining 5 of 16 studies on ND-GBM patients assessed the predictive power in terms of the OS and PFS of the tensor isotropic and anisotropic components (respectively p and q) [24,51,68,80,82]. In a study by Mohsen et al. and two by Yan et al. [51,68,82], volumetric analyses were performed on postoperative p and q maps. Mohsen et al. [51], considering visive abnormality volumes on p and q maps, assessed that a minimal invasive pattern predicts a higher PFS. Similarly, the size of the abnormal q regions was correlated with OS and PFS in studies by Yan et al. [68,82]. Li et al., in two separate studies [24,80], evaluated the association with survival outcomes of the histogram and joint histogram features from the normalized DTI-p and q maps values within the enhancing and non-enhancing tumour zones. In the first study, the joint histogram features, in particular the proportion of the non-enhancing tumour subregion with the decreased isotropic diffusion and increased anisotropic diffusion, were associated with OS and PFS in multivariate models [24]. Similar results on q were found for histogram analysis performed in the second study by Li et al. [80]. In the same study, the p and q histogram metrics showed a significantly incremental value in predicting OS and PFS with respect to the clinical variables alone.

Only Paldino et al. [46], in a longitudinal study, investigated the survival predictive power of the DTI metrics in R-GBM patients. In particular, changes in the MD mean and FA between pre- and post-treatment were investigated in the T1 contrast-enhancing zones and abnormalities on FLAIR images, resulting in significant results in terms of OS and PFS only for changes in the MD mean within abnormalities on FLAIR images.

#### 3.3.3. Other Models Metrics

Only three of the selected studies investigated the association with survival outcomes of the diffusion parameters arising from models different from DWI an DTI [25,26,28]. Puig et al. [25] investigated IVIM metrics within contrast-enhancing and non-enhancing regions, finding that the pseudo-diffusion coefficient and perfusion fraction in the contrast-enhancing regions were significant predictors of 6 months survival. Krishnan et al. [28] performed a survival analysis of the volumetric and intensities metrics arising from the RSI diffusion technique, comparing them with those arising from DWI. While none of ADC metrics were associated with OS and PFS, the RSI volume fraction and 90th percentile within the FLAIR hyperintensity tumour regions were associated with PFS and OS. Lastly, Chakhoyan et al. [26] showed that, in comparison with ADC (which show significant value in predicting OS), any diffusion metrics arising from the DKI and SE models provided additional prognostic value.

### 3.4. Quality Assessment

**Figure 3:**
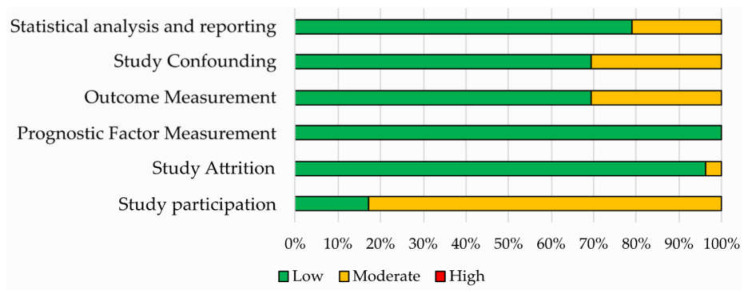
Risk of bias assessment according to the six domains of the Quality in Prognostic Studies (QUIPS) tool for the 52 studies included in the systematic review.

Results of the QUIPS assessment are shown in Figure 3 and reported in Supplementary Materials Table S3. The risk of bias was ranked low or moderate across all the studies for all the six QUIPS domains. Most studies displayed a low risk of bias in the domains of study attrition, outcome measurement, study confounding and statistical analysis and reporting. All studies were judged to be at low risk of bias for study attrition, while a higher percentage of studies with moderate risk of bias was found concerning the study participation domain.

## 4. Discussions

In this systematic review, we aimed at investigating the role of the diffusion MR biomarkers in predicting survival outcomes in GBM patients. In the last decade, although diffusion imaging is not yet a primary modality in GBM management, an ever-growing number of studies aimed at investigating the role of diffusion MR metrics in OS and PFS prediction. This is mainly due to the ability of the diffusion MRI techniques (with respect to traditional morphological MRI) of providing a quantitative assessment of intratumoral heterogeneity of GBM infiltration, which is of clinical significance for targeted surgery and therapy, and are crucial for improving GBM patient survival. However, the role of diffusion MRI metrics in prediction and evaluation of survival outcomes has not been fully addressed and results are often controversial or unsatisfactory. In this scenario, our systematic review can provide important new insights and help to reach a common view on the use of diffusion MRI metrics for GBM prognosis. After appropriate inclusion and exclusion criteria, we examined 52 studies from 2010 onwards, evaluating the association of diffusion MRI metrics with OS and PFS in ND-GBM and R-GBM patients. We found that the most investigated diffusion metrics were those associated with DWI and DTI, and only few studies considered different diffusion models (non-Gaussian DWI models and RSI). The main findings and conclusions of the selected studies varied from each other, often showing inconsistencies and not a clear idea about the actual usefulness and the effective prognostic power of the diffusion MR biomarkers.

The most investigated metrics were the minimum and mean ADC value and its histogram metrics arising from uni- or bi-variate histogram analysis for DWI, and MD and FA for DTI, either as mean values or as histogram parameters. These metrics, obtained from different tumour zones (e.g., T1 contrast enhancement and non-enhancement and FLAIR signal abnormality), showed prognostic significance in a different study setting for both ND- and R-GBM patients [54,59,63,66,69,76]. However, there is no shortage of studies showing adverse results [12,47,57,64]. A lower number of studies investigated metrics arising from different approaches, such as four-component histogram analysis, fDM maps analysis, radiomics analysis and DTI isotropic and anisotropic components [24,43,49,51,68,74,80,82,83].

The most interesting finding emerged from our analysis is that, in most studies, the diffusion metrics could not be identified as independent prognostic parameters besides established clinical factors (like age, KPS, MGMT promoter methylation status and extent of resection) and other quantitative parameters arising from other MRI techniques (such as rCBV from perfusion MRI or DCE–MRI-derived metrics), also performing analysis of radiomic features [62,72,74,76,83,84]. In this context, it should be mentioned that the recently introduced radiomics approach may contribute significantly towards survival prediction and stratification of GBM patients [74,83], even if further studies specific for GBM patients are required.

Characteristics of the included studies, such as patient treatment, study aim and setting, diffusion sequence parameters, areas on which ROIs were placed, diffusion metrics for the same diffusion model, analysis methods and the OS and PFS definition, were highly variable across studies, preventing us from performing a meta-analysis. Among the just-mentioned sources of variability, segmentation approaches used for ROI delineation remained tricky considering that the T1-contrast enhancement sequence highlights more perfused tumoral regions, reducing the intratumoral heterogeneity sampling. On the other side, ROI outlining based on FLAIR sequences are affected by peritumoural oedema that can reduce the significance of the findings due to the inclusion of unaffected components in the analyses [19,85]. The variability in patient treatment is particularly noticeable in studies on R-GBM patients, and this is largely due to the lack of a standard of care and the limited efficacy of the therapeutic options, which also justify the larger presence of clinical trials among studies on R-GBM than those on ND-GBM [86].

Moreover, since most of included studies were retrospective, they are supposed to have more bias and should be validated through prospective studies [87].

To our knowledge, this is the first systematic review aiming at summarizing the role of diffusion MR imaging metrics in predicting survival in GBM patients. To date, systematic studies exploring diffusion metrics applied to GBM patients aimed at evaluating the power of DWI and DTI for differentiating GBM from brain metastasis or PCNSL, or from lower grade gliomas [32,33,88]. However, analysis of survival outcomes was out of the purpose of these works. Oltra-Sastre et al. [89] systematically reviewed the multiparametric MR imaging biomarkers associated with clinical outcomes, including the survival outcomes. However, this study was not focused on diffusion metrics and considered studies on gliomas of any WHO grade.

Nowadays, the greatest drawback of diffusion MRI techniques is the lack of standardization [23,90,91]. Continued research efforts and standardization of acquisition parameters and analytic methods, possibly with automation, are required to arrive at the most effective approach that can be applied across institutions. Moreover, due to the ever-growing application of artificial intelligence techniques in the context of medical imaging, it could be interesting to further explore the association between diffusion metrics and GBM survival outcomes using machine learning and deep learning techniques, as done by Chang et al. and Petrova et al. for DWI metrics [65,78] or by Zacharaki et al. and Nie et al. [92,93] for DTI metrics, even if the latter two works were not specific for GBM patients.

## 5. Conclusions

In conclusion, although further work is required to optimize the methodology associated with the acquisition and analysis of diffusion MR parameters, it seems that diffusion metrics may improve the prediction of survival outcomes in GBM patients, in particular in combination with clinical parameters and conventional or radiomic imaging features arising from multimodal techniques.

## Figures and Tables

**Table 1:** Characteristics of the included studies. Abbreviations: P/R = Prospective/Retrospective; DWI = Diffusion Weighted Imaging; DTI = Diffusion Tensor Imaging; DKI = Diffusion Kurtosis Imaging; IVIM = Intravoxel Incoherent Motion; SE = Stretched Exponential; RSI = Restriction Spectrum Imaging; SO = Survival Outcome; ROI = Region of Interest; OS = Overall Survival; PFS = Progression-Free Survival; NAWM = Normal Appearing White Matter; CEL = Contrast Enhancing Lesion; NEL = Non-Enhancing Lesion; EOR = Extent of Resection; KPS = Karnofsky Performance Score; 2-GMM = 2 Gaussian Mixture Model; 4-GMM = 4 Gaussian Mixture Model; ND/R = Newly Diagnosed/Recurrent; RT = Radio Therapy; TMZ = Temozolomide; BV = Bevacizumab; FS = Field Strength; EPI = Echo Planar Imaging; FOV = Field of View; TR = Relaxation Time; TE = Echi Time; Seq = Sequence; ST = Slice Thickness.

Author	Year	Np	Study Design (P/R)	Diagnosis (ND/R)	Treatment	MRI Timepoints	Diffusion Model/s	Diffusion Acquisition Details	Diffusion Metrics Investigated	SO	ROI Info	Survival Analysis	Main Findings
Saksena et al. [11]	2010	34	R	ND	Surgery/biopsy; RT; Chemotherapy	Preoperative	DTI	FS = 3 T, Seq.= EPI, FOV = 128 × 128 mm, TR = 17 ms, TE = 84.3 ms, ST = 5 mm, no gape, NEX = 1, gradients applied in 25 non collinear directions, b = 0, 1000 s/mm^2^	FA, ADC, CL, CP, and CS (mean and min)	PFS	ROIs on FLAIR signal abnormality in CEL and NEL; manual segmentation.	Kaplan–Meier survival curves; univariate and multivariate Cox proportional hazards models adjusted for age, KPS, EOR.	Univariate analysis showed that min values of FA, MD, CP, CS were associated with PFS rate. The multivariate analysis demonstrated that only min CP was a PFS predictor.
Li et al. [41]	2011	64	P	ND	Surgery/biopsy; RT; Chemotherapy: 23 TMZ, 29 TMZ with tarceva, 10 poly ICLC, 2 R115777	Postoperative: pre-treatment, post-treatment	DWI	FS = 3 T or 1.5T, Directions: 3–6, Seq. = EPI, TR/TE = 5000–10,000/63–110 ms, matrix 128 × 128 or 256 × 256, ST 3–5 mm, 21–40 slices, b 0–1000 s/mm^2^.	nADC median and percentiles (pre-RT, changes between preRT and post-RT)	OS, PFS	ROIs on NAWM, CEL, T2 hyperintensity lesions, and the NE lesion; automatic segmentation	Kaplan–Meier survival curves; Univariate and multivariate Cox proportional hazards models adjusted for age and field strength.	No diffusion parameters associated with OS or PFS in univariate analysis; lower nADC in CEL and higher nADC in T2 hyperintensity lesion and NEL associated with worse OS in multivariate analysis.
Pope et al. [42]	2011	121	R	ND	Surgery/biopsy; RT; Chemotherapy post resection: 59 TMZ and BV, 62 TMZ. Chemotherapy on recurrence: 34 add BV.	Preoperative	DWI	FS = 1.5 T, Seq. = EPI, section thickness of 3–5 mm, FOV of 24 cm, matrix size: 256 × 256 for most patients. b = 0, 1000 s/mm^2^.	ADC_L (2-GMM histogram)	OS, PFS	ROIs on T1CEL; semiautomatic segmentation.	Kaplan–Meier with log-rank and Wilcoxon test; uni- and multivariate Cox regression models with RPA class and MGMT methylation status	ADC values did not stratify OS and PFS in the control group; pretreatment ADC histogram analysis can stratify PFS in BV-treated patients with newly diagnosed GBM.
Ellingson et al. [43]	2012	143	R	ND	Surgery/biopsy; RT; Chemotherapy: TMZ	Postoperative: pretreatment, posttreatment	DWI	FS = 1.5 T, Seq. = EPI, TE/TR = 102.2/8000 ms, NEX = 1, ST = 5 mm, gap = 1 mm, matrix size = 128 × 128, FOV = 24 cm using a twice-refocused epi, b = 0 s/mm^2^ and b = 1000 s/mm^2^	fDM metrics	OS, PFS	ROIs on regions of FLAIR signal abnormality and T1 CEL; segmentation method not specified	Log-rank analysis of Kaplan–Meier curves; Cox-regression analysis adjusted for age and KPS	Patients exhibiting a large volume of tissue with decreased ADC are statistically more likely to have a short PFS and OS.
Romano et al. [44]	2012	47	R	ND	Surgery; RT; Chemotherapy: TMZ following by adjuvant tmz therapy.	Preoperative	DWI	FS = 1.5 T, Seq. = EPI, b-values 0, 500, and 1000 s/mm^2^, ST = 5 mm; TR= 3000 ms; TE = 84 ms; gap, = 0.3mm; matrix = 256 × 256 mm, acquisition time = 1.40 min.	ADC min	OS, PFS	ROIs on T1CEL; semiautomatic segmentation.	Kaplan–Meier, log rank, uni- and multivariate Cox regression models with MGMT methylation status	patients with high ADCmin values have higher OS and PFS than patients with low ADCmin values.
Pope et al. [45]	2012	97	R	R	Treatments pre-recurrence: RT and TMZ; Chemotherapy on recurrence: BV or BV and CPT-11 (Irinotecan)	Pretreatment	DWI	NR	ADC_L and LCP (2-GMM histogram)	6-PFS, overall PFS, OS	ROIs on T1CEL; semiautomatic segmentation.	The Kaplan–Meier method with log-rank test, uni- and multivariate Cox models adjusted for age and enhancing tumour volume at recurrence	ADC-L was predictive for 6-PFS, OS and PFS. LCP alone was only predictive of 6-PFS.
Paldino et al. [46]	2012	15	P	R	Treatments pre-recurrence: RT and TMZ; Chemotherapy on recurrence: BV and Irinotecan	Pretreatment, posttreatment	DTI	FS = 1.5 T, Seq. = EPI, TR/TE = 6000/100 ms; flip angle, 90 degrees; 4 NEX; matrix = 128 × 128; voxel size 1.72 × 1.72 × 5 mm	Changes in MD and FA mean	OS, PFS	ROIs on T1 CEL and abnormalities on FLAIR images; semiautomatic segmentation	Cox proportional hazard model.	Patients with a change in MD within FLAIR signal abnormality region had significantly shorter OS and PFS than those with no change.
Zikou et al. [47]	2012	17	P	ND	Surgery; RT; Chemotherapy: TMZ.	Preoperative	DTI	FS = 1.5 T, Seq. = EPI, TR:9807 ms, TE:131 ms, FOV:230 mm, acquisition matrix: 128 × 128, slice thickness: 3 mm, max b-value: 700 s/mm^2^, 16 non-collinear diffusion directions	normalized MD and FA	OS	ROIs on T1CEL; manual segmentation	Log-rank analysis of Kaplan–Meier curves; Multivariate Cox regression analysis not performed due to statistical non-significance.	No significant correlation was found between MD, FA and OS.
Sunwoo et al. [48]	2013	26	R	ND	Surgery/biopsy; RT; Chemotherapy: TMZ.	Preoperative	DWI	FS = 1.5 T, TR/TE = 6000/63 (at b 0 and 1000 s/mm^2^, 25 sections, bandwidth of 1953Hz/voxel, ST 5 mm, gap 1, FOV = 240 × 240 mm, a matrix = 160 × 160, voxel resolution 1.5 × 1.5 × 5.0 mm, directions 3.	ADC mean, ADC 5th percentile (histogram)	PFS	ROIs on T1CEL; manual segmentation	Kaplan–Meier	A positive significant relationship was demonstrated between PFS and the mean ADC. 5th percentile was not significantly associated with PFS.
Ellingson et al. [49]	2013	143	R	ND	Surgery/biopsy; RT; Chemotherapy: TMZ	Postoperative: pretreatment, posttreatment	DWI	FS = 1.5 T, TE/TR = 102.2 ms/8000 ms, NEX = 1, ST = 5 mm, gap = 1, matrix size = 128 × 128, FOV = 24 cm using a twice-refocused EPI, b 1000 s/mm^2^ and b 0 s/mm^2^.	prob-fDM metrics	OS, PFS	ROIs on regions of FLAIR signal abnormality; semiautomatic segmentation	Log-rank analysis of Kaplan–Meier curves	Patients with a large volume fraction of tumour showing a decrease in ADC through prob-fDM had a significantly shorter PFS and OS.
Nakamura et al. [50]	2013	138	R	ND	Surgery/biopsy; RT; Chemotherapy	Preoperative	DWI	FS = 1.5 T, Seq. = EPI, TE/TR = 3600/81 ms, ST = 5, gap = 1 mm, 128 × 128 matrix, 230 mm FOV, one acquisition, b = 1000 s/mm^2^	ADC min	OS, PFS	ROIs on T1CEL; manual segmentation	Log-rank analysis of Kaplan–Meier curves; multivariate Cox regression analysis with age, KPS and surgery/biopsy	Tumours with low ADC min are associated with low PFS and OS.
Mohsen et al. [51]	2013	25	R	ND	Surgery; RT; Chemotherapy: TMZ.	Preoperative or immediately postoperative	DTI	FS = 1.5 T, Seq. = EPI, TR/TE: 12 k/95 ms, ST/inter-slice gap: 4/4 mm, resolution 256 × 256, 25 directions, two b = 0, 1000 s/mm^2^ FOV: 24 × 24 cm.	p and q maps pattern (diffuse, localised or minimally invasive	PFS	ROIs on the visible abnormality on p and q maps; manual segmentation	Log-rank analysis of Kaplan–Meier curves; univariate Cox regression analysis	Invasiveness of DTI pattern was associated with PFS. A minimal invasive pattern predicts a higher PFS.
Ellingson et al. [52]	2013	132	R	R	Chemotherapy: 89 patients BV; 43 variety of chemotherapies but never exposed to BV	Pretreatment	DWI	FS = 1.5 or 3 T, Seq. = EPI, TE/TR = 80–110 ms/4–10 s, 1 average, section thickness = 5 mm with gap = 1 mm, matrix size = 128 × 128, and FOV = 22–25 cm, b = 1000 and b 0 s/mm^2^.	ADC_L (2-GMM histogram)	OS, PFS	ROIs on T1CEL; semiautomatic segmentation	Log-rank analysis of Kaplan–Meier curves; univariate and multivariate Cox regression analysis adjusted for age, treatment cohort	Patients with lower ADC_L had a significantly longer PFS and OS compared with those having higher ADC_L.
Omuro et al. [53]	2014	40	P	ND	Surgery/biopsy; RT; Chemotherapy: TMZ and BV	Postoperative (pretreatment)	DWI	NR	nADC mean	PFS, 1y-OS	ROIs on T1 CEL; manual segmentation	Log-rank analysis of Kaplan–Meier curves	Lower baseline ADC was associated with prolonged OS, but not PFS.
Rahman et al. [54]	2014	91	R	R	Treatments pre-recurrence: standard radiation and TMZ therapy; Chemotherapy on recurrence: BV	Pretreatment	DWI	FS = 1.5, Seq. = monopolar EPI, TE/TR = 80–110 ms/4–10 ms, ST = 5 mm, gap = 1 mm, matrix size = 128 × 128 mm, FOV = 22–25 cm, b value 1000 and 0 s/mm^2^	%ADC_L, %ADC_H, and ADC_L/ADC_M (4-GMM histogram)	OS, PFS	ROIs on T1 CEL NE T2/FLAIR abnormality; automatic segmentation	Kaplan–Meier curves; uni- and multivariable analysis with Cox proportional hazards model adjusted for clinical variables	Baseline ADC_L/ADC_M within NE T2/FLAIR volume and ADC_H within T1 CEL can stratify OS and PFS.
Wen at al. [55]	2015	36	R	ND	Surgery/biopsy; RT; Chemotherapy: TMZ, erlotinib and BV.	Postoperative: pretreatment and posttreatment at 1 month, 2 months and every 2 months (up to a maximum of 14 months)	DWI	FS = 3 T, b = 1000 (dir = 6, NEX = 4) and ADC maps were calculated using in-house developed software.	ADC percentiles (histogram); 2-GMM histogram metrics; fDM metrics	OS, PFS	ROIs on T1 CEL and T2/FLAIR hyperintensity; semiautomatic segmentation	Kaplan–Meier curves; Univariate and multivariate Cox regression analysis adjusted for age, KPS, EOR	ADC10% within the T2L at 2 months was strongly associated with OS and PFS. fDM metrics showed an association with OS and PFS within the CEL when considered by univariate analysis, but not in the T2L.
Coban et al. [12]	2015	58	R	ND	Surgery; RT; Chemotherapy	Preoperative	DWI	FS = 3 T, Seq. = EPI, acceleration factor of 2, FOV = 22 × 22 cm^2^; b 0, 1000 s/mm^2^, section thickness = 3 mm; number of sections = 40; acquisition time = 8 min.	ADC min	15 months OS	ROIs on T1 CEL and visually low ADC; manual segmentation	ROC analysis, Kaplan–Meier curves	ADC min was not useful for differentiating patients having short or long survival.
Elson et al. [56]	2015	52	R	ND	Surgery; RT; Chemotherapy	Postoperative	DWI	NR	ADCmean, ADCmin, nADCmean, nADCmin	OS, PFS	ROIs on hyperintense T2/FLAIR; manual segmentation	Log-rank analysis on Kaplan–Meier data; multivariate Cox regression analysis adjusted for age, EOR, KPS	Regression analysis indicated that normalized ADC values provide the strongest association with PFS and OS.
Lee et al. [57]	2015	24	R	ND	Surgery; RT; Chemotherapy: TMZ	Postoperative	DWI	FS = 3 T, Seq. = EPI, b-values of 0 and 1000 s/mm^2^, three orthogonal directions.	nADC (histogram metrics)	PFS	ROIs on T1 CEL; manual segmentation	Log-rank analysis on Kaplan–Meier data	nADC not associated with PFS
Zhang et al. [58]	2015	52	R	R	Treatments pre-recurrence: surgical resection + RT + TMZ; Chemotherapy on recurrence: BV	Pretreatment; 2 posttreatment scans	DWI	FS = 1.5 or 3 T, Seq. = EPI, b = 0 and 3 diffusion-weighted acquisitions with b 1000 s/mm^2^	low-ADC volume and percent change, normalized 5th percentile low ADC values and percent changes	OS	ROIs on T1 CEL and FLAIR hyperintense abnormality corresponding to low ADC signal; manual segmentation	Kaplan–Meier curves; uni- and multivariate Cox regression analysis with clinical and imaging metrics	At the second post-BV scan, the volume of the low-ADC lesion was inversely associated with OS. Normalized 5th percentile low-ADC value and its percent change were not associated with OS.
Jamjoom et al. [59]	2015	46	R	ND	No surgery; 4 treatment groups	Preoperative	DTI	FS = 3 T, Seq. = EPI, Acceleration factor of 2, b = 0 and b = 1000, six directions, TR = 2435–4813 ms, TE = 48–62 ms, voxel size 1.6 × 1.6 × 5 mm, FOV = 230 × 180 × 159 mm. 15 directions, TR = 3175–8000 ms, TE = 57–90 ms, voxel size 2 × 2 × 3.3 mm, FOV 224 × 224 × 105 mm	MDmin (from MD map); histogram metrics (from MD gradient maps)	OS	ROIs on T1 CEL that visually appeared dark on the MD maps; semiautomatic segmentation	Univariate and multivariate Cox regression analysis adjusted for treatment protocol and gender	Lower minMD and higher MD gradient values for the 10th and 75th percentile of the tumour boundary predict short OS.
Wen at al. [60]	2015	75	R	ND	Surgery/biopsy; 44 RT; Chemotherapy: TMZ and enzastaur, 31 TMZ + erlotinib and BV	Postoperative: pretreatment, posttreatment (after 1, 2 and 4 months)	DTI	FS = 3 T, six-directional, Seq. = DWI, b = 1000 s/mm^2^, number of excitations = 4.	MD, FA and longitudinal and radial eigenvalues (histogram metrics)	OS, PFS	ROIs on T1 CEL and T2 hyperintense lesions; manual segmentation	Log-rank analysis on Kaplan–Meier data; multivariate Cox regression analysis adjusted for age, EOR, KPS	For the TMZ + enza cohort: volumes of regions with low MD values at 1-month scan associated with OS and at 2-month scan associated with PFS. For the TMZ + erl + bev cohort, volumetric diffusion parameters and MD and EVrad were associated with OS and PFS at different timepoints.
Chang et al. [61]	2015	120	R	ND	Surgery/biopsy; RT; Chemotherapy: TMZ following by adjuvant tmz therapy.	Postoperative	DWI	FS = 1.5 T, TE/TR = 80–120 ms/5000 ms, matrix size = 128 128, ST = 3 mm with no interslice gap, and b-values of 0 and 1000 s/mm^2^ in three orthogonal directions.	ADC_L, ADC_H (2-GMM histogram)	OS, PFS	ROIs on CEL on T1 subtraction images; segmentation method not specified	Log-rank analysis on Kaplan–Meier data and multivariate Cox regression analysis adjusted for age	Patients with lower ADC_L have shorter OS and PFS. ADC_H was not predictive.
Burth et al. [62]	2016	125	R	ND	Surgery/biopsy; Radiotherapy and Chemotherapy: 5 different treatment regimens	Preoperative	DWI	FS = 3 T, TR = 5300 ms, TE = 90 ms, b 0 and b 1200, pixel size 1.769 mm/1.769 mm, image matrix 130 × 130, ST 5 mm, flip angle 908, FoV = 229 × 229 mm.	ADC histogram metrics	OS, PFS	ROIs on T1 CEL and T2/FLAIR hyperintensity; semiautomatic segmentation	Univariate and multivariable Cox regression analyses including age, sex, EOR, KPS, rCBV	Univariate analysis showed that 10th percentile ADC in CEL and T2/FLAIR were significantly associated with OS, but not with PFS. In multivariable analysis diffusion-derived MRI parameters did not predict survival.
Shankar et al. [63]	2016	84	R	ND	Surgery/biopsy; RT; Chemotherapy: TMZ	Preoperative	DWI	FS = 1.5 T, Seq. = EPI. TR = 8000 ms, TE = 73.6 ms, FOV = 260 mm, matrix size = 160 × 192, section ST = 5 mm, gap = 1.5 mm, b = 0 and b = 1000 in three orthogonal directions.	nADC min	OS	Whole tumour volume identified on T1 CEL and FLAIR; restricted diffusion ROIs identified on ADC map; manual segmentation	Log-rank analysis on Kaplan–Meier data and multivariate Cox regression analysis	Positive association between nADC min and OS.
Van der Hoorn et al. [64]	2016	14	R	ND	Surgery; RT; Chemotherapy: TMZ and adjuvant TMZ	Postoperative: preradiotherapy, postradiotherapy	DWI	FS = 1.5 T, Seq. = EPI, TR/TE = 6000–12,500/64–108 ms; flip angle 90°; FOV 220–300 × 220–300 mm; 52–66 slices; 0–4 mm slice gap; voxel size 0.86–1.2 × 0.86–1.2 × 4–5 mm, b-value of 0 and 1000 s/mm^2^, scanned in 3–25 directions.	nADC histogram metrics	OS, PFS	ROIs automatically segmented in periresectional area and manually adjusted.	Univariate and multivariate Cox regression analysis adjusted for age and MGMT methylation status	The increase in ADC value postradiotherapy in comparison to preradiotherapy did not predict an increase in PFS or OS neither in univariate nor multivariate analysis.
Chang et al. [65]	2016	126	R	R	Treatments pre-recurrence: surgical resection + RT + TMZ; Chemotherapy on recurrence: BV	Pretreatment, posttreatment	DWI	Seq. = Monopolar EPI, TE/TR = 80–110 ms/4–10 s, section thickness = 5 mm, gap = 1 mm, matrix size = 128 × 128 mm, FOV= 22–25 cm, b-value 1000 and 0 s/mm^2^.	ADC (texture, 4-GMM histogram metrics)	OS, PFS	ROIs on T1 CEL, T2/FLAIR; semiautomatic segmentation	Machine-learning predictive model based on random-forest and including conventional MRI and DWI metrics	Model based on multiparametric MRI imaging metrics (of which DWI) was able to predict OS
Zolal et al. [39]	2016	31	R	R	Surgery; RT; Chemotherapy: TMZ	Preoperative (prior to second surgery)	DWI	FS = 1.5 T, b = 0 and 1000 s/mm^2^, ST of 5 mm, and voxel sizes between 0.9 and 2 mm.	ADC histogram metrics	OS, PFS, Survival after 2nd surgery	ROIs in T1 CEL (manual selection or semi-automated adaptive thresholding)	Log-rank analysis on Kaplan–Meier data and multivariate Cox regression analysis including also age, EOR, tumour size	ADC histogram skewness associated with OS and PFS in univariate analysis and with survival after 2^nd^ surgery in multivariate analysis.
Choi et al. [66]	2016	112	R	ND	Surgery; RT; Chemotherapy: TMZ.	Preoperative	DTI	FS = 3 T, b values of 600 s/mm^2^ and 0 s/mm^2^, 32 directions, FOV = 8413.4/77; 220 mm; section thickness = 2 mm, matrix = 112 × 3 × 112.	MD histogram metrics	12-OS, 16-OS, 12-PFS	ROIs on T1 CEL; semiautomatic segmentation	Log-rank analysis on Kaplan–Meier data and multivariate Cox regression analysis with MGMT methylation status, age, KPS, EOR	At univariate analysis, lower MD histogram parameters were significant predictors of poor OS and PFS; Multivariable models with MD parameters had significantly higher performances that those without MD parameters for OS and PFS prediction.
Huber et al. [67]	2016	122	R	ND	Surgery; RT; Chemotherapy: TMZ.	Preoperative	DTI	FS = 3 T, DTI direction 15 or 6 directions	mean ADC, FA	OS	ROIs in the CEL, central region (CR), and the FLAIR-hyperintense NE peritumoral region	Kaplan–Meier curves; multivariate Cox regression analysis with age, KPS, tumour volume, infiltration	Patients with low FA values in CEL showed a significantly improved OS in univariate analysis. In multivariate analysis FA values could not be identified as independent prognostic parameters besides clinical factors.
Yan et al. [68]	2016	31	R	ND	Surgery; Chemotherapy: TMZ.	Preoperative, postoperative	DTI	FS = 3 T, Seq. = EPI, TR/TE = 8300/98 ms; flip angle 90°; FOV 192 × 192 mm; 63 slices; no slice gap; and voxel size 2 × 2 × 2 mm, b-values = 0, 350, 650, 1000, 1300, and 1600 s/mm^2^, 13 directions.	EOR (extent of resection) based on p and q maps	OS, PFS	ROIs representing EOR manually placed on T1	Univariate and multivariate Cox regression analysis including age, MGMT methylation status, IDH-1 mutation, tumour volume and location	larger residual abnormal q volume predicted significantly shorter PFS; larger resection of abnormal q area improved OS.
Puig et al. [25]	2016	15	P	ND	Surgery; RT; Chemotherapy: TMZ	Preoperative	IVIM MRI	FS = 1.5, Seq. = EPI, slice = 24, TR = 3000 ms, TE = 76 ms, EPI factor was 41, FOV = 200 mm, section thickness = 5 mm, matrix = 96 × 77 mm, pixel size = 2.4 × 2.9 × 5 mm.13 b-values: 0, 10, 20, 30, 50, 100, 150, 200, 350, 500, 650, 800, and 1000 s/mm^2^, acquisition time was 3 min 48 s per patient.	D, D*, f	OS	ROIs in T1 CEL and NEL; manual segmentation	Kaplan–Meier curves; multivariate Cox regression analysis with clinical and DSC metrics	f and D* in CEL are associated with 6 months survival
Kondo et al. [69]	2017	76	R	ND	NS	Preoperative	DWI	NR	L-ADC_L, B-ADC_L, B&L-ADC_L (2-GMM histogram)	OS, PFS	ROIs in T1 CEL manual segmentation	Kaplan–Meier curves; univariate Cox regression analysis	B&L-ADCL was strongly associated with poor PFS and OS
Krishnan et al. [28]	2017	45	R	ND	Surgery	Postoperative (pretreatment)	DWI, RSI	FS = 3 T, Seq. = EPI, TE/TR = 96 ms/17 ms, FOV = 24 cm, matrix = 96 × 966 × 48, voxel size = 2.5 mm, 4 b-values (b 0, 500, 1500, and 4000 s/mm^2^, 6 and 15 unique diffusion directions for each nonzero b-value, respectively 8 min scan time.	ADC and RSI volume fraction, 10th and 90th percentile	OS, PFS	3D ROIs on T1 CEL and FLAIR hyperintensity; semiautomatic segmentation	Univariate and multivariate Cox regression analysis combined with age, gender and resection type	No ADC metrics were associated with PFS and OS. RSI volume fraction was associated with PFS and OS, RSI 90th percentile associated with OS.
Ellingson et al. [70]	2017	258	R	R	Chemotherapy: 5 different regimens	Pretreatment	DWI	FS = 1.5 or 3 T, Seq. = monopolar EPI, TE/TR = 80–110 ms/4–10 s, NEX = 1, ST = 5 with 0–1 mm interslice gap, matrix size = 128 × 128, FOV = 220–256 mm. b = 0 and b = 1000 s/mm^2^.	ADC_L (2-GMM histogram)	OS	3D ROIs on T1 subtraction maps; semiautomatic segmentation	Log-rank analysis and multivariate Cox regression analysis including age, enhancing tumour volume	Pretreatment ADC_L was an independent predictive biomarker for OS in anti-VEGF therapies, but not in lomustine.
Galla et al. [71]	2017	65	R	R	Chemotherapy: BV	Pretreatment, posttreatment	DWI	FS = 3 T, Seq. = EPI, FOV= 24 × 24 cm^2^, b= 0, 1000 s/mm^2^, ST = 5 mm.	changes in nADC mean and min	OS	ROIs on ADC maps corresponding to the T1 CEL and NEL; manual segmentation	Cox regression analysis adjusted for age, tumour size, prior treatments	The change in mean ADC was significantly associated with OS. The change in min ADC in the NEL was not associated with OS
Boonzaier et al. [72]	2017	43	R	ND	Surgery; RT; Chemotherapy	Preoperative	DTI	FS = 3 T, Seq. EPI, TR/TE = 8300/98; flip angle = 90°; FOV = 192 × 3 × 192 mm; 63 sections; no section gap; voxel size = 2.0 × 3.2 × 3.2 mm; b values 0–1000 s/mm^2^.	volumetric analysis: ADC low volume	OS, PFS	ROIs in CEL and NEL regions based on ADC-rCBV maps; manual segmentation	Log-rank analysis and multivariate Cox regression accounting for age, CE volume, IDH-1 mutation, MGMT methylation status, EOR	Volumetric analysis of ADC-rCBV ROIs in NEL helps in stratifying PFS and OS
Heiland et al. [73]	2017	21	P	ND	Surgery	Preoperative	DTI	NR	AD, RD, mean MD and FA	OS, PFS	ROIs on the whole CEL; manual segmentation	The Kaplan–Meier, univariate Cox-Regression tests	Patients with high MD in the contrast-enhancing region had a significantly better OS. Patients with low FA in the contrast-enhancing region had a significantly better OS.
Chakhoyan et al. [26]	2018	23	P	ND	Surgery; RT; Chemotherapy: TMZ	Postoperative: pretreatment, posttreatment	DWI, DKI, SE	FS = 3 T, Seq. EPI, TR/TE = 13,400/103 ms, a flip angle of 90°, 52 contiguous slices, ST = 3 mm, no inter-slice gap, an in-plane resolution of 2 mm × 2 mm, matrix = 128 × 128 mm, b values: 0, 50, 100, 250, 500, 750, 1000, 2500, 3500 and 5000 s/mm^2^. The total acquisition time for the DWI scan was 6 min.	ADC, K, D, DDC, alpha	OS	ROIs placed in NAWM and CEL; semiautomatic segmentation	Log-rank analysis on Kaplan–Meier curves and multivariate Cox regression analysis including age, MGMT status, tumour volume at baseline	ADC show significant value in predicting OS. DKI and SE metrics did not show significant value in OS prediction.
Li et al. [24]	2018	115	P	ND	Surgery; Adjuvant therapy postoperative	Preoperative	DTI	NR	Joint histogram features from Normalized DTI-p and q maps	OS, PFS	ROIs in CEL and NEL; manual segmentation	Kaplan–Meier, Cox regression accounting for IDH-1 mutation, MGMT methylation status, sex, age, EOR	joint histogram features were associated with OS and PFS and improved survival model performance.
Bae et al. [74]	2018	217	R	ND	Surgery; RT; Chemotherapy: TMZ	Preoperative	DTI	FS = 3 T, b values 600 and 0 s/mm^2^, 32 directions, FOV = 8413.4/77; 220 mm; section thickness = 2 mm; matrix 112 × 3 × 112.	Radiomic features	OS, PFS	ROIs in necrosis, CEL, NEL on T2/FLAIR; semiautomatic segmentation	Random Survival Forest prediction model from multiparametric MRI	Radiomic prediction model including clinical and multiparametric MRI metrics (of which DTI metrics) was able to predict OS and PFS
Rulseh et al. [75]	2019	43	R	ND	Surgery; RT; Chemotherapy: TMZ	Preoperative	DWI	NR	ADC histogram metrics	OS, PFS	Whole-brain masks generated using FSL	The Kaplan–Meier, univariate Cox-Regression analysis	Median ADC was the best variablefor PFS prediction, while p85 was the best variable for OS prediction.
Kim et al. [76]	2019	93	R	ND	Surgery/biopsy; RT; Chemotherapy: TMZ	Preoperative	DWI	FS = 1.5 T, Seq. = EPI, TR/TE 3000 ms/80 ms; FOV = 240 × 240 mm; matrix = 164 × 162; ST = 5 mm; gap = 1 mm, b value 0 and 1000 s/mm^2^.	ADC histogram metrics	OS	ROIs on T1CE and FLAIR; manual segmentation	Log-rank test on Kaplan–Meier curves, unsupervised K-means clustering	ADC histogram parameters demonstrated a significant association with OS.
Buemi et al. [77]	2019	17	R	R	Chemotherapy: 13 BV, 4 fotemustine and BV	Pretreatment, posttreatment	DWI	FS = 1.5 T, Seq = EPI, TE/TR = 90 ms/1000 ms, NEX 2, slice thickness 5 mm with 1 mm interslice distance, matrix size = 320 × 320 mm, and FOV = RL 240 mm, AP 282 mm, FH 131 mm, b = 0 and b = 1000 s/mm^2^.	ADC_L, ADC_H (2-GMM histogram)	OS, PFS	ROIs on T1 CEL and T2/FLAIR abnormalities; semiautomatic segmentation	Kaplan–Meier with log-rank test, multivariate Cox regression adjusted for clinical variables	In univariate analysis, ADC_L in CEL was significantly predictive of PFS and OS. In multivariate analysis, the ADC_L was predictive for PSF but not OS.
Petrova et al. [78]	2019	54	R	R	Chemotherapy: RT and TMZ Chemotherapy on recurrence: BV	Pretreatment	DWI	FS = 1.5 T or 3 T, b = 0 and b = 1000, matrix = 128 × 128, FOV = 22–24 cm with a ST of 5 mm.	ADC histogram metrics	OS, 6PFS	ROIs on T1 CE; manual segmentation	6 machine learning classifiers	Diffusion and perfusion imaging using an SVM was able to predict 6PFS. Less power was shown to predict OS.
Flores-Alvarez et al. [79]	2019	36	R	ND	Surgery; RT; Chemotherapy	Preoperative	DTI	FS = 3 T, FOV of 22 × 22 mm^2^, b-value of 1000 s/mm^2^, 25 directions, TR = 10.000 ms, TE = 101.8 ms, ST of 3 mm and a Matrix array of 112 × 112.	FA	OS	ROIs in necrosis, CEL, oedema, normal controlateral, peritumoral oedema; manual segmentation	Log-rank test on Kaplan–Meier curves	Significant association between the peritumoral oedema measurement of FA with intervals of OS.
Li et al. [80]	2019	80	P	ND	Surgery/biopsy; RT (17.4%, 20/115); Chemotherapy: TMZ (73.0%, 84); Best supportive care (9.6%, 11/115);	Preoperative	DTI	NR	histogram analysis of normalized DTI-p and q maps	OS, PFS	ROIs in CEL and NEL; manual segmentation	Kaplan–Meier and Cox regression accounting for IDH-1, mutation, MGMT methylation status, sex, age, EOR, tumour volume	A higher mean value of anisotropic diffusion (q) in NE region was associated with worse OS and PFS. 5 p and q histogram metrics showed significantly incremental value in predicting 12-month OS and PFS.
Mohan et al. [81]	2019	48	R	ND	Surgery; RT; Chemotherapy: TMZ. Other therapies: BV (8 patients)	Preoperative	DTI	FS = 3 T, Seq. = Epi, parallel acquisition (GRAPPA), acceleration factor of 2.30 Directions, a b-value of 1000 s/mm^2^, a total acquisition time of 8 min, TR/TE 5000/86 ms, NEX = 3, FOV = 22 × 22 cm^2^, slice thickness = 3 mm, number of sections = 40.	mean FA, MD	OS	ROIs in CC if there were CE lesions on T1 or signal abnormality on T2 and FLAIR; manual segmentation	Kaplan–Meier with log-rank test, multivariate Cox regression adjusted for clinical variables	FA from the invaded CC was positively correlated with OS.
Yan et al. [82]	2019	51	R	ND	Surgery; RT; Chemotherapy: TMZ.	Preoperative, postoperative	DTI	FS = 3 T, Seq. = EPI, TR/TE = 8300/98 ms; flip angle 90°; FOV = 192 mm^2^; 63 slices; no slice gap; voxel size 2 mm^3^, b values (0, 350, 650, 1000, 1300 and 1600 s/mm^2^), scanned in 12 directions.	EOR (extent of resection) based on p and q maps	OS, PFS	ROIs representing EOR on pre-MR; manual segmentation	Kaplan–Meier with log-rank test	Larger abnormal q regions showed better PFS and OS
Park et al. [83]	2020	248	R	ND	Surgery; RT; 60 Gy Chemotherapy: TMZ	Preoperative	DWI	FS = 3 T, Seq. = EPI, TR/TE = 3000/56 ms; b = 0 and b = 1000 s/mm^2^, FOV= 25 cm; ST/gap = 5 mm/2 mm; matrix = 256 × 256; acquisition time, 39 s.	radiomic features	OS	ROIs on T1 CEL; semiautomatic segmentation	Log-rank test on Kaplan–Meier curves; radiomics predictive models	Multiparametric MR model (incorporating also ADC features) was able to predict OS
Song et al. [84]	2020	19	R	R	Before recurrence: RT + TMZ Chemotherapy on recurrence: immune checkpoint inhibitors (ICIs)	Pretreatment, posttreatment	DWI	FS = 3 T, Seq. = EPI, TR/TE = 4025/82 ms, b value 0 and 1000 s/mm^2^.	nADC, changes between pre and post treatment	PFS6	ROIs on T1 CEL; semiautomatic segmentation	Univariate analysis	nADC was able to assess PFS6
Patel et al. [40]	2020	67	R	R	35 treated with BV; 35 repeated surgery Chemotherapy: 19% BV monotherapy; 81% BV + TMZ and small molecular inhibitors.	Pretreatment	DWI	FS = 1.5 or 3 T, Seq. = EPI, ST = 3 mm with no interslice gap, b-values of 0, 500 and 1000 s/mm^2^) or diffusion tensor imaging with 64 directions.	ADC_L (2-GMM histogram)	OS	ROIs on T1 CEL; manual segmentation	Log-rank analyses on Kaplan–Meier data and Cox proportional hazard models adjusted for age, tumour volume	ADCL was an independent predictor of OS in the BV cohort, but not the surgical cohort.

## Supplementary Materials

The following are available online at https://www.mdpi.com/2072-6694/12/10/2858/s1, Table S1: Key terms used in literature search, Table S2: PRISMA Checklist, Table S3: Quality assessment using QUIPS tool.
