# Supplementary material for: Predicting Survival in Glioblastoma Patients Using Diffusion MR Imaging Metrics—A Systematic Review
Source: Cancers (Basel). 2020 Oct 4;12(10):2858. doi: 10.3390/cancers12102858 (PMC7600641; doi:10.3390/cancers12102858)
Supplement: Supplementary file 1 [file cancers-12-02858-s001.pdf]

Supplementary Materials

Table 1. Key terms used in literature search.

- "(Glioblastoma OR GBM) AND diffusion imaging AND survival"
- "(Glioblastoma OR GBM) AND diffusion mri AND survival"
- "(Glioblastoma OR GBM) AND apparent diffusion coefficient AND survival"
- "(Glioblastoma OR GBM) AND adc AND survival"
- "(Glioblastoma OR GBM) AND diffusion model AND survival"
- "(Glioblastoma OR GBM) AND diffusion kurtosis imaging AND survival"
- "(Glioblastoma OR GBM) AND DKI AND survival"
- "(Glioblastoma OR GBM) AND IVIM AND survival"
- "(Glioblastoma OR GBM) AND intravoxel incoherent motion AND survival"
- "(Glioblastoma OR GBM) AND stretched exponential AND survival"
- "(Glioblastoma OR GBM) AND diffusion imaging AND overall survival"
- "(Glioblastoma OR GBM) AND diffusion mri AND overall survival"
- "(Glioblastoma OR GBM) AND apparent diffusion AND coefficient AND overall survival"
- "(Glioblastoma OR GBM) AND adc AND overall survival"
- "(Glioblastoma OR GBM) AND diffusion model AND overall survival"
- "(Glioblastoma OR GBM) AND diffusion kurtosis imaging AND overall survival"
- "(Glioblastoma OR GBM) AND DKI AND overall survival"
- "(Glioblastoma OR GBM) AND IVIM AND overall survival"
- "(Glioblastoma OR GBM) AND intravoxel incoherent motion AND overall survival"
- "(Glioblastoma OR GBM) AND stretched exponential AND overall survival"
- "(Glioblastoma OR GBM) AND diffusion imaging AND progression free survival"
- "(Glioblastoma OR GBM) AND diffusion mri AND progression free survival"
- "(Glioblastoma OR GBM) AND apparent diffusion AND coefficient AND progression free survival"
- "(Glioblastoma OR GBM) AND adc AND progression free survival"
- "(Glioblastoma OR GBM) AND diffusion model AND progression free survival"
- "(Glioblastoma OR GBM) AND diffusion kurtosis imaging AND progression free survival"
- "(Glioblastoma OR GBM) AND DKI AND progression free survival"
- "(Glioblastoma OR GBM) AND IVIM AND progression free survival"
- "(Glioblastoma OR GBM) AND intravoxel incoherent motion AND progression free survival"
- "(Glioblastoma OR GBM) AND stretched exponential AND progression free survival"

Predicting survival in Glioblastoma patients using Diffusion MR imaging metrics – a systematic review.

Table 2. PRISMA Checklist.

Section/topic	#	Checklist item	Reported on page #
**TITLE**			
Title	1	Identify the report as a systematic review, meta-analysis, or both.	1
**ABSTRACT**			
Structured summary	2	Provide a structured summary including, as applicable: background; objectives; data sources; study eligibility criteria, participants, and interventions; study appraisal and synthesis methods; results; limitations; conclusions and implications of key findings; systematic review registration number.	1
**INTRODUCTION**			
Rationale	3	Describe the rationale for the review in the context of what is already known.	1-3
Objectives	4	Provide an explicit statement of questions being addressed with reference to participants, interventions, comparisons, outcomes, and study design (PICOS).	2-3
**METHODS**			
Protocol and registration	5	Indicate if a review protocol exists, if and where it can be accessed (e.g., Web address), and, if available, provide registration information including registration number.	3
Eligibility criteria	6	Specify study characteristics (e.g., PICOS, length of follow-up) and report characteristics (e.g., years considered, language, publication status) used as criteria for eligibility, giving rationale.	3
Information sources	7	Describe all information sources (e.g., databases with dates of coverage, contact with study authors to identify additional studies) in the search and date last searched.	3
Search	8	Present full electronic search strategy for at least one database, including any limits used, such that it could be repeated.	3
Study selection	9	State the process for selecting studies (i.e., screening, eligibility, included in systematic review, and, if applicable, included in the meta-analysis).	3
Data collection process	10	Describe method of data extraction from reports (e.g., piloted forms, independently, in duplicate) and any processes for obtaining and confirming data from investigators.	3
Data items	11	List and define all variables for which data were sought (e.g., PICOS, funding sources) and any assumptions and simplifications made.	3
Risk of bias in individual studies	12	Describe methods used for assessing risk of bias of individual studies (including specification of whether this was done at the study or outcome level), and how this information is to be used in any data synthesis.	4
Summary measures	13	State the principal summary measures (e.g., risk ratio, difference in means).	NA
Synthesis of results	14	Describe the methods of handling data and combining results of studies, if done, including measures of consistency (e.g., I^2) for each meta-analysis.	NA

Predicting survival in Glioblastoma patients using Diffusion MR imaging metrics – a systematic review.

Section/topic	#	Checklist item	Reported on page #
Risk of bias across studies	15	Specify any assessment of risk of bias that may affect the cumulative evidence (e.g., publication bias, selective reporting within studies).	NA
Additional analyses	16	Describe methods of additional analyses (e.g., sensitivity or subgroup analyses, meta-regression), if done, indicating which were pre-specified.	NA
**RESULTS**			
Study selection	17	Give numbers of studies screened, assessed for eligibility, and included in the review, with reasons for exclusions at each stage, ideally with a flow diagram.	4
Study characteristics	18	For each study, present characteristics for which data were extracted (e.g., study size, PICOS, follow-up period) and provide the citations.	5-23
Risk of bias within studies	19	Present data on risk of bias of each study and, if available, any outcome level assessment (see item 12).	23
Results of individual studies	20	For all outcomes considered (benefits or harms), present, for each study: (a) simple summary data for each intervention group (b) effect estimates and confidence intervals, ideally with a forest plot.	NA
Synthesis of results	21	Present results of each meta-analysis done, including confidence intervals and measures of consistency.	NA
Risk of bias across studies	22	Present results of any assessment of risk of bias across studies (see Item 15).	NA
Additional analysis	23	Give results of additional analyses, if done (e.g., sensitivity or subgroup analyses, meta-regression [see Item 16]).	NA
**DISCUSSION**			
Summary of evidence	24	Summarize the main findings including the strength of evidence for each main outcome; consider their relevance to key groups (e.g., healthcare providers, users, and policy makers).	23-24
Limitations	25	Discuss limitations at study and outcome level (e.g., risk of bias), and at review-level (e.g., incomplete retrieval of identified research, reporting bias).	24
Conclusions	26	Provide a general interpretation of the results in the context of other evidence, and implications for future research.	25
**FUNDING**			
Funding	27	Describe sources of funding for the systematic review and other support (e.g., supply of data); role of funders for the systematic review.	25

From: Moher D, Liberati A, Tetzlaff J, Altman DG, The PRISMA Group (2009). Preferred Reporting Items for Systematic Reviews and Meta-Analyses: The PRISMA Statement. PLoS Med 6(7): e1000097. doi:10.1371/journal.pmed1000097

Predicting survival in Glioblastoma patients using Diffusion MR imaging metrics – a systematic review.

Table 3. Quality assessment using QUIPS tool.

Study	Year	Study participation	Study Attrition	Prognostic Factor Measurement	Outcome Measurement	Study Confounding	Statistical Analysis and Reporting
Saksena et al.	2010	Moderate	Moderate	Low	Low	Low	Low
Li et al.	2011	Low	Low	Low	Low	Low	Low
Pope et al.	2011	Moderate	Low	Low	Low	Low	Low
Ellingson et al.	2012	Moderate	Low	Low	Low	Low	Low
Romano et al.	2012	Moderate	Low	Low	Low	Low	Low
Pope et al.	2012	Moderate	Low	Low	Low	Low	Low
Paldino et al.	2012	Low	Low	Low	Low	Low	Low
Zikou et al.	2013	Low	Low	Low	Low	Moderate	Moderate
Sunwoo et al.	2013	Moderate	Low	Low	Low	Moderate	Moderate
Ellingson et al.	2013	Moderate	Low	Low	Low	Moderate	Moderate
Nakamura et al.	2013	Moderate	Low	Low	Low	Low	Low
Mohsen	2013	Moderate	Low	Low	Low	Moderate	Moderate
Ellingson et al.	2013	Moderate	Low	Low	Moderate	Low	Low
Omuro et al.	2014	Low	Low	Low	Low	Moderate	Moderate
Rahman	2015	Moderate	Low	Low	Low	Low	Low
Wen at al.	2015	Moderate	Low	Low	Low	Low	Low
Coban et al.	2015	Moderate	Low	Low	Low	Moderate	Moderate
Elson et al.	2015	Moderate	Low	Low	Low	Low	Low
Lee et al.	2015	Moderate	Low	Low	Low	Moderate	Moderate
Zhang et al.	2015	Moderate	Low	Low	Low	Low	Low
Jamjoom et al.	2015	Moderate	Low	Low	Moderate	Low	Low
Wen at al.	2016	Moderate	Low	Low	Low	Low	Low
Chang et al.	2016	Moderate	Low	Low	Low	Low	Low
Burth et al.	2016	Moderate	Low	Low	Low	Low	Low
Shankar et al.	2016	Moderate	Low	Low	Low	Low	Low
Van der Hoorn et al.	2016	Moderate	Low	Low	Low	Low	Low
Chang et al.	2016	Moderate	Low	Low	Low	Moderate	Low
Zolal et al.	2016	Moderate	Low	Low	Low	Low	Low
Choi et al.	2016	Moderate	Low	Low	Low	Low	Low
Huber et al.	2016	Moderate	Low	Low	Low	Low	Low
Yan et al.	2016	Moderate	Low	Low	Moderate	Low	Low
Puig et al.	2017	Low	Low	Low	Low	Low	Low
Kondo et al.	2017	Moderate	Low	Low	Low	Low	Low
Krishnan et al.	2017	Moderate	Low	Low	Low	Low	Low

Ellingson et al.	2017	Moderate	Low	Low	Moderate	Low	Low
Galla et al.	2017	Moderate	Low	Low	Moderate	Low	Low
Boonzaier et al.	2017	Moderate	Low	Low	Moderate	Low	Low
Heiland et al.	2018	Low	Moderate	Low	Moderate	Moderate	Moderate
Chakhoyan et al.	2018	Low	Low	Low	Moderate	Low	Low
Li et al.	2018	Low	Low	Low	Moderate	Low	Low
Bae et al.	2019	Moderate	Low	Low	Moderate	Moderate	Low
Rulseh et al.	2019	Moderate	Low	Low	Moderate	Moderate	Moderate
Kim	2019	Moderate	Low	Low	Moderate	Moderate	Low
Buemi et al.	2019	Moderate	Low	Low	Low	Low	Low
Petrova et al.	2019	Moderate	Low	Low	Moderate	Moderate	Low
Flores-Alvarez et al.	2019	Moderate	Low	Low	Low	Low	Low
Li et al.	2019	Low	Low	Low	Moderate	Low	Low
Mohan et al.	2019	Moderate	Low	Low	Low	Low	Low
Yan et al.	2020	Moderate	Low	Low	Moderate	Moderate	Moderate
Park et al.	2020	Moderate	Low	Low	Low	Moderate	Low
Song et al.	2020	Moderate	Low	Low	Low	Moderate	Moderate
Patel et al.	2020	Moderate	Low	Low	Moderate	Low	Low
